# Protein interactions at the higher plant nuclear envelope: evidence for a linker of nucleoskeleton and cytoskeleton complex

**DOI:** 10.3389/fpls.2014.00183

**Published:** 2014-05-07

**Authors:** David E. Evans, Vidya Pawar, Sarah J. Smith, Katja Graumann

**Affiliations:** Department of Biological and Medical Sciences, Oxford Brookes UniversityOxford, UK

**Keywords:** nuclear envelope, SUN domain proteins, KASH domain proteins, nuclear structure, LINC complex

## Abstract

Following the description of SAD1/UNC84 (SUN) domain proteins in higher plants, evidence has rapidly increased that plants contain a functional linker of nucleoskeleton and cytoskeleton (LINC) complex bridging the nuclear envelope (NE). While the SUN domain proteins appear to be highly conserved across kingdoms, other elements of the complex are not and some key components and interactions remain to be identified. This mini review examines components of the LINC complex, including proteins of the SUN domain family and recently identified plant Klarsicht/Anc/Syne-1 homology (KASH) domain proteins. First of these to be described were WIPs (WPP domain interacting proteins), which act as protein anchors in the outer NE. The plant KASH homologs are C-terminally anchored membrane proteins with the extreme C-terminus located in the nuclear periplasm; AtWIPs contain a highly conserved X-VPT motif at the C-terminus in contrast to PPPX in opisthokonts. The role of the LINC complex in organisms with a cell wall, and description of further LINC complex components will be considered, together with other potential plant-specific functions.

## INTRODUCTION

The nuclear envelope (NE) fulfills many important functions: protecting and enclosing the genetic material, facilitating transport, involvement in cell signaling, and providing physical and structural bridges. These functions of support, transport, and communication are required to different extents in various organisms from single cells to complex metazoans and require a high level of complexity. This is achieved by differentiation of the proteins of the inner and outer nuclear membranes (INM and ONM, respectively), each having a unique protein composition providing specific cytoplasm-facing and nucleoplasm-facing functions.

Communication across the NE occurs through protein bridges that link across the periplasmic space between the INM and ONM. These protein bridges have multiple functions, providing support and anchorage for the genetic material and nucleoskeleton, positioning, and moving the nucleus and acting as a pathway of signaling. While these functions and structures are conserved in eukaryotes, there are marked differences in the proteins that are involved (see below and Zhou and Meier, 2013). This mini review will consider advances in the identification of the proteins of the higher plant NE with a focus on proteins that bridge the membrane.

## BRIDGING THE NUCLEAR PERIPLASM; THE LINC COMPLEX

**FIGURE 1:**
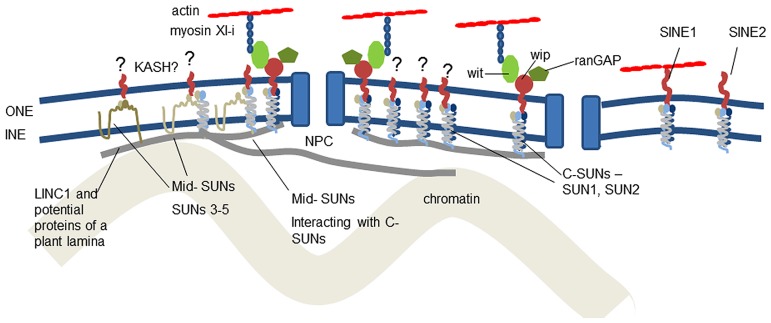
**Location of known and suggested proteins at the plant nuclear envelope.** It is now clear that C-SUN domain proteins (SUNs1–2) in the inner nuclear membrane interact with proteins of the outer nuclear membrane and the nucleoskeleton. The role of mid-SUNs (SUNs 3–5) remains to be elucidated, but they also interact with C-SUNs and may also constitute a key component of anchorages for the cytoskeleton, nucleoskeleton, and nuclear pores. Anchorage of SINE, Wit, Wip, and RanGAP all depend on SUN interaction. The plant LINC complexes are involved in two actin-NE linkages – the direct SINE1-actin connection and the WIP-WIT-myosin XI-I – actin linkage.

Micrographs of the nucleus reveal many connections between structural proteins of the cytoplasm and nucleoplasm, the nuclear membrane and the nuclear pores (Fiserova et al., 2009). The membranes are separated by a space of about 50 nm and it is in this space that interactions between ONM and INM proteins occur. The major bridge in this space is the linker of nucleoskeleton and cytoskeleton (LINC) complex (Crisp et al., 2006). It is conserved across eukaryotes and has remarkable diversity of function through modification of two families of constituent proteins. It provides anchorage for NE-associated proteins of the nucleoskeleton and cytoskeleton, which support, move and shape the NE and chromatin and position the nuclear pores (**Figure 1**).

The LINC complex has two key constituents, the SAD1/UNC84 (SUN) domain proteins of the INM and the Klarsicht/Anc/Syne-1 homology (KASH) domain proteins of the ONM (Crisp et al., 2006). They attach by the interaction of the SUN and KASH domains located at the extreme C-termini of the respective proteins. In most opisthokonts, there are two ubiquitously expressed SUN domain proteins (as well as other family members with more specialized expression patterns); while KASH domain proteins are far more diverse in structure and function.

## SUN DOMAIN PROTEINS

The name SUN domain derives from UNC84, described by Malone et al. (1999) in *Caenorhabditis elegans* embryos and Sad1, a spindle pole body component in *Schizosaccharomyces pombe* (Hagan and Yanagida, 1995). Both contain a C-terminal SUN-domain. Initial studies revealed two ubiquitously expressed homologs in opisthokonts which are type II integral membrane proteins. The C-terminal SUN domain is located in the nuclear periplasm, with the N-terminus in the nucleoplasm where it interacts with B-type lamins (Crisp et al., 2006). A coiled coil domain is located between the transmembrane domain and the SUN domain in the nuclear periplasm. While the C-terminal SUN domain is highly conserved, the N-terminus is not; the lamin binding domain is absent in *HsSUN3, 4,* and *5* (Lee et al., 2002; Fridkin et al., 2004; Haque et al., 2006). The crystal structure of human SUN2 indicates that proteins with a classical C-terminal SUN domain form trimers (Sosa et al., 2013; Zhou and Meier, 2013). These in turn bind three KASH domains; binding is further strengthened by interaction between conserved cysteines forming a disulphide bridge. The majority of the bridge between the two membranes is formed by the SUN trimer with the KASH domain lying very close to the ONM (Sosa et al., 2012).

SAD1/UNC84 domain proteins are involved in multiple cellular functions. The yeast SUN domain proteins Sad1 (*S. pombe*) and Mps3 (*Saccharomyces cerevisiae*) are located at spindle pole bodies (SPBs) and the INM (Tran et al., 2001; Bupp et al., 2007). Specifically, SpSad1 and ScMps3 are localized to the half bridge, part of the central plaque of the SPB associated with the NE (Hagan and Yanagida, 1995; Jaspersen et al., 2006). As well as the ubiquitously expressed SUN1 and 2, mammals contain three SUN domain proteins (SUN3, 4, and 5) which are tissue specific in distribution (Razafsky and Hodzic, 2009). Some SUN domain proteins are expressed differentially during development; for instance, *C. elegans* SUN1 is expressed in the germ line and early embryo, while UNC84 is found in adult somatic cells and embryos after the 24-cell stage (Lee et al., 2002; Fridkin et al., 2004).

## PLANT SUN DOMAIN PROTEINS – THE EVIDENCE

First descriptions for homologs of plant SUN domain proteins were of SpSad1 in *Arabidopsis thaliana* by van Damme et al. (2004), and *Oryza sativa* by Moriguchi et al. (2005) and indicated location at the phragmoplast and mitotic spindle. The significance of the higher plant SUN domain proteins was overlooked until the first detailed characterisation by Graumann et al. (2010). This study revealed AtSUN1 and AtSUN2 to be localized to the NE in interphase and provided the first evidence of components of a putative LINC complex in plants.

Genomic sequencing reveals that proteins with a classical C-terminal SUN domain are present throughout the kingdom plantae, including the club moss *Selaginella moellendorffii,* the moss *Physcomitrella patens,* algae, and monocot and dicot species. They have been characterized in detail in the dicot *A. thaliana* (Graumann et al., 2010; Graumann and Evans, 2011; Oda and Fukuda, 2011) and the monocot *Zea mays* (Murphy et al., 2010). Each species has two proteins with a C-terminal SUN domain (AtSUN1 and AtSUN2; Graumann et al., 2010, and ZmSUN1 and ZmSUN2; Murphy et al., 2010). AtSUN1 and 2 show a higher degree of homology (68% identity, 1.00 E^-178^) with each other than with either ZmSUN1 or 2 (41%, 4.0 E^-79^ and 2.0 E^-70^, respectively) suggesting separate gene duplication events. Some difference in function and binding as well as location is suggested for the different SUN proteins. The two plant C-terminal SUN domain proteins are significantly smaller than mammalian and closest in size to yeast Sad1. There is a strong structural resemblance with a single coiled coil domain located between the N-terminal transmembrane domain and the C-terminal SUN domain (Graumann and Evans, 2010; Graumann et al., 2010).

Recently, in addition to interaction with putative plant KASH domain proteins in the outer NE (see below), Graumann (2014) has demonstrated that plant SUN domain proteins interact with putative nucleoskeletal proteins of the NMCP/LINC family (Ciska and Moreno, 2013, 2014). Specifically, the N-terminus of AtSUN1 and 2 can interact with LINC1 and immobilize it at the nuclear periphery (Graumann, 2014). In the absence of lamins in plants, these SUN–LINC interactions are a first indicator of SUN-nucleoskeletal anchorage in plants.

Studies of higher plants reveal an additional family of proteins containing a central SUN domain in addition to the C-terminal SUNs (Murphy et al., 2010). Genome analysis of maize indicates three of these mid-SUN proteins; two that are ubiquitously expressed (ZmSUN3 and 4), and one which is pollen specific (ZmSUN5). These plant mid-SUN proteins are located at the nuclear periphery (Murphy et al., 2010). They have also been described in opisthokonts; the best studied being osteopotentia (Opt), which is a rough endoplasmic reticulum (rER) resident (Sohaskey et al., 2010). Opt is suggested to function as a biomechanical adaptor stabilizing the rER by interacting with the cytoskeleton. The second mid-SUN protein to be explored in opisthokonts was yeast SUN like protein (SLP1; Friederichs et al., 2012). In their study the authors showed that SLP1 is co-localized in cortical and perinuclear ER with a 65 kDa ER membrane protein. While it does not interact directly with the C-terminal SUN protein, Mps3, it affects its localisation at the NE. Friederichs et al. (2012) therefore hypothesize a role for the mid-SUN protein in the maintenance of the Mps3 pool at the NE. Whether the SUN proteins with a central SUN domain are involved in nucleo-cytoplasmic bridging, in plants or opisthokonts, remains unclear but is beginning to be investigated.

## KASH DOMAIN PROTEINS; MULTI-FUNCTIONAL COMPONENTS OF THE LINC COMPLEX

The term KASH domain derives from members of a family of proteins, described in *D. melanogaster* (Klarsicht), *C elegans,* (ANC-1; Starr and Han, 2002) and mammals (Syne-1 and 2, also known as Nesprin 1 and 2; Apel et al., 2000) which interact with SUN domain proteins (Starr and Fridolfsson, 2010). They are generally transmembrane proteins located at the ONM, with a conserved C-terminal KASH domain in the perinuclear space close to a transmembrane domain. Starr (2009) proposed four criteria to characterize them: location at the ONM; KASH domain mediated interaction with the SUN domain; ONM localisation dependent on the SUN–KASH domain interaction (Crisp et al., 2006); and a non-conserved, cytoplasmic N-terminal domain that interacts with cytoskeletal proteins like actin and dynein.

The KASH domain includes a transmembrane domain and a short stretch of amino acids (typically between 9 and 35) in the periplasm ending in a conserved sequence which in most animal KASH proteins is PPPX (Razafsky and Hodzic, 2009; Starr and Fridolfsson, 2010). KASH domain proteins vary widely in size; the largest being the 1300 kDa protein Msp-300/nesprin. KASH domain proteins have an N-terminal cytoskeletal binding domain separated from a single transmembrane domain by a series of spectrin repeats or coiled coils (Lenne et al., 2000). Like SUN, KASH-domain proteins form homomers (Djinovic-Carugo et al., 2002; Mislow et al., 2002). Therefore, a SUN–KASH complex comprises three SUN domain proteins (as a homo- or hetero-trimer) each associated with a KASH domain protein which may be associated with other adjacent KASH domain proteins (**Figure 1**). Binding occurs when the PPPT motif of the KASH domain fits into a hydrophobic pocket formed by the SUN domain trimer. Specifically, the penultimate proline appears important for this binding and is widely conserved (Razafsky and Hodzic, 2009).

Metazoan nesprins vary widely in size and interactions. Largest are the proteins formed by the *Syne1/Nesp1* gene that encodes a range of splice isoforms, the biggest being the 1000 kDa Nesprin 1 Giant (Nesp1G). Nesp1G has an N-terminal actin binding domain, made up of two calponin homology domains. *Nesprin 2* (also called *Syne2/NUANCE*) encodes Nesp2G of 800 kDa also with an N-terminal cytoplasmic actin binding domain (Apel et al., 2000; Zhang et al., 2002; Zhen et al., 2002). Nesprin 3, 100 kDa, lacks an actin binding domain, but binds intermediate filaments via an interaction with plectin; while Nesprin 4 is smaller (42 kDa) and interacts with the cytoskeleton via kinesin. Some smaller nesprins co-localize and interact with the INM protein, emerin (Mislow et al., 2002; Zhang et al., 2005; Wheeler et al., 2007).

Klarsicht/Anc/Syne-1 homology domain proteins also have a role in controlling nuclear size in non-plant systems (Lu et al., 2012). Nesprin 1 and 2 interact with Nesprin 3 so that both ends contact the nuclear surface, first via their C-terminal KASH-domains and second by interaction between their N-termini and Nesprin 3. Nesprin interaction with the cytoskeleton also forms a lattice-like filamentous network covering the ONM (Lu et al., 2012).

## PLANT KASH DOMAIN PROTEINS

The search for plant KASH domain proteins took longer than that for the SUN domain proteins. The breakthrough came from the realization that a well-studied plant NE protein showed characteristics similar to the KASH domain proteins and the subsequent demonstration of its interaction with SUN domain proteins in a collaboration between the Graumann and Meier laboratories (Zhou et al., 2012).

Previous work (Xu et al., 2007) had identified an NE-associated *Arabidopsis* family of the WPP (tryptophan-proline-proline) domain proteins. These include WPP1, WPP2, and RanGTPase-activating protein 1 (RanGAP1) and are characterized by the presence of various repeats of the WPP motif in the protein sequence (Meier, 2000; Rose and Meier, 2001). These had been shown to be localized to the NE by interactions with two plant-specific protein families – the WIPs (WPP domain interacting proteins) and the WITs (WPP interacting tail anchored proteins). WIPs and WITs oligomerise to provide the anchorage of the WPP proteins. For instance, by anchoring RanGAP1 to the NE (Xu et al., 2007), they are involved in generating the RanGTP gradient necessary for transport through the nuclear pores and hence nucleo-cytoplasmic transport. Both WIPs and WITs are C-terminally anchored membrane proteins with the C-terminus located in the periplasm. However, only the AtWIPs were found to contain a highly conserved X-VPT motif (X, hydrophobic amino acid; Zhou et al., 2012). This motif contains a penultimate proline, similar to the opisthokont PPPX, and is conserved in other plant species. Furthermore, Zhou et al. (2012) had also shown that deletion of this extreme C-terminal VVPT of AtWIP1 reduced its NE localization. Thus, the overall domain structure and localisation of AtWIPs made them good candidates for plant KASH proteins.

Confirmation of the WIPs as KASH domain proteins depended on demonstration of their SUN interaction and that the SUN binding was indispensable to their NE localisation. Interaction of the VVPT motif with SUN domain proteins was shown by a number of means. Deletion of all, or parts of, the SUN domain abolished interaction of AtWIP1, 2, and 3 in a pull down assay with AtSUN2 (Zhou et al., 2012). Also, fluorescence recovery after photobleaching (FRAP) assays revealed significant increase in mobility of AtWIPs after deletion of the VVPT domain. Zhou et al. (2012) further demonstrated that in a *sun1-KO/ sun2-KD* mutant transformed to express GFP-AtWIP1, the fluorescent signal from the WIP protein was predominantly cytoplasmic, resembling the distribution shown for a WIP1 truncation mutant in which the C-terminal VVPT had been deleted, confirming the requirement for the SUN domain protein for nuclear localisation. Comparison of the AtWIP1 C-terminus shows a low degree of similarity to opisthokont KASH domains. It is small in size, though similar to *C. elegans* ZYG-12B and KDP-1 and *D. discoideum* Interaptin (Xiong et al., 2008; McGee et al., 2009; Minn et al., 2009). The penultimate proline is highly conserved, as is the terminal serine/threonine.

The location of RanGAP at the pore complex was the first *bona fide* function identified for a plant LINC complex; it differs from mammalian systems, where RanGAP anchorage occurs by direct attachment to the nuclear pores by RanBP2. Further functions of the plant LINC complex are being elucidated. Tamura et al. (2013) used a myosin XI-I mutant of Arabidopsis, kaku1, to explore attachment of the cytoskeleton to the NE. They were able to show that myosin XI-I localizes at the NE and attaches to WIT1, which interact with WIP proteins attached to the ONM. Plant nuclei move in a number of circumstances, including in response to blue light and fungal infection, and are known to involve an actin-rather than a microtubule-based system (Nagai, 1993; Skalamera and Heath, 1998). Tamura et al. (2013) showed that the myosin XI-I attached to the LINC complex interacts with long cables of actin in the plant cytoplasm, thus enabling long distance movement of the nucleus, using blue-light as a model for nuclear migration. Also, down-regulation of WIP and WIT proteins results in an increase in the sphericity of the nucleus. Thus, the WIP-WIT-myosin XI-I extension of the LINC complex is multi-functional in the NE, attaching the LINC complex to elements of the actin cytoskeleton while governing other functions like nuclear sphericity. However, it is clear that they are not the only proteins involved as WIP, WIT, and Kaku1 mutants do not show altered development, although nuclear positioning and mobility are essential for development.

In fact, a very recent study by Zhou et al. (2014) identified a second group of plant KASH proteins – the sun interacting NE (SINE) proteins. These proteins are also C-terminally anchored, contain a short periplasmic tail and the C-terminal X-VPT motif. Similar to the WIPs, SINE–SUN interactions are mediated by the VPT and SUN motifs and the NE localisation of SINEs is dependent on the presence of SUNs (Zhou et al., 2014). Functional analysis of SINE mutants revealed that SINE2 is involved in the innate immunity response to oomycete pathogens while SINE1 is involved in nuclei positioning in guard cells. Indeed, SINE1 is the first plant KASH protein identified to be tissue-specifically expressed. It has an armadillo repeat domain, with which it links to the actin cytoskeleton (Zhou et al., 2014). Hence, in addition to the myosin XI-I – WIT–WIP linkage, a second more direct actin association with KASH proteins occurs in plants.

Other interactions at the plant NE seem to be implicated in the interaction between the nucleus and microtubule cytoskeleton. The mini review of Batzenschlager et al. (2014) in this research topic presents evidence for γ-tubulin complex protein 3 (GCP3)-interacting proteins (GIPs) by recruiting γ-tubulin complexes (γ-TuCs) in interaction with the microtubule cytoskeleton in interphase. The plant ONM is known to act as MTOC due to the association of the γ-TuCs with the NE. While it remains unclear whether and how plant LINC complexes are involved in NE-MTOC associations, the putative role of GIP/MZT1 at the NE offers a new line of enquiry into NE-microtubule cytoskeleton associations (Batzenschlager et al., 2014).

## CONCLUSION AND FUTURE PROSPECTS

A role for the LINC complex in both nuclear shape and movement indicates this is a highly conserved and fundamental system of nuclear attachment. In the absence of a variety of other NE proteins found in ophistokonts, the plant SUN and KASH proteins take on an increased importance. Future work to elucidate the functions of the C-SUNs and the mid-SUNs, together with identification of further plant KASH proteins and exploration of their roles is of great importance as the system is central to many key plant cell processes from control of gene expression to chromosome segregation in meiosis.

## Conflict of Interest Statement

The authors declare that the research was conducted in the absence of any commercial or financial relationships that could be construed as a potential conflict of interest.
